# Multi-Index Driver Drowsiness Detection Method Based on Driver’s Facial Recognition Using Haar Features and Histograms of Oriented Gradients

**DOI:** 10.3390/s24175683

**Published:** 2024-08-31

**Authors:** Eduardo Quiles-Cucarella, Julio Cano-Bernet, Lucas Santos-Fernández, Carlos Roldán-Blay, Carlos Roldán-Porta

**Affiliations:** 1 Instituto de Automática e Informática Industrial, Universitat Politècnica de València, Camino de Vera, s/n, 46022 Valencia, Spain; jucaber@etsii.upv.es (J.C.-B.); lusanfe1@etsid.upv.es (L.S.-F.); 2 Institute for Energy Engineering, Universitat Politècnica de València, Camino de Vera, s/n, edificio 8E, Escalera F, 5ª planta, 46022 Valencia, Spain; carrolbl@die.upv.es (C.R.-B.); croldan@die.upv.es (C.R.-P.)

**Keywords:** driver drowsiness detection, driver monitoring, biometric information, facial expressions, artificial vision, Haar features, histograms of oriented gradients

## Abstract

It is estimated that 10% to 20% of road accidents are related to fatigue, with accidents caused by drowsiness up to twice as deadly as those caused by other factors. In order to reduce these numbers, strategies such as advertising campaigns, the implementation of driving recorders in vehicles used for road transport of goods and passengers, or the use of drowsiness detection systems in cars have been implemented. Within the scope of the latter area, the technologies used are diverse. They can be based on the measurement of signals such as steering wheel movement, vehicle position on the road, or driver monitoring. Driver monitoring is a technology that has been exploited little so far and can be implemented in many different approaches. This work addresses the evaluation of a multidimensional drowsiness index based on the recording of facial expressions, gaze direction, and head position and studies the feasibility of its implementation in a low-cost electronic package. Specifically, the aim is to determine the driver’s state by monitoring their facial expressions, such as the frequency of blinking, yawning, eye-opening, gaze direction, and head position. For this purpose, an algorithm capable of detecting drowsiness has been developed. Two approaches are compared: Facial recognition based on Haar features and facial recognition based on Histograms of Oriented Gradients (HOG). The implementation has been carried out on a Raspberry Pi, a low-cost device that allows the creation of a prototype that can detect drowsiness and interact with peripherals such as cameras or speakers. The results show that the proposed multi-index methodology performs better in detecting drowsiness than algorithms based on one-index detection.

## 1. Introduction

Road transport is currently the most widely used transport for long distances and short daily trips in the freight transport sector. The accidents during these journeys are known as commuting accidents and constitute a specific source of risk at the wheel.

Driving fatigue is a significant factor in many road crashes (10–20%) [1]. The number of fatalities involving a drowsy driver was 1.9% of total fatalities in the USA in 2019 [2]. Based on a USA crash analysis, references [3,4] estimated that 13% of crashes in which a person was hospitalised and 21% of crashes in which a person was killed involved a drowsy driver.

The National Highway Traffic Safety Administration (NHTSA) estimated that drowsy driving accounted for 91,000 traffic accidents, which caused approximately 50,000 injuries and 800 deaths, as reported by the police in 2017 [5]. However, individuals in traffic safety, sleep science, and public health have unanimously agreed that these figures underestimate the impact of drowsy driving. The National Sleep Foundation reports that 54% of adult drivers feel drowsy while driving. In addition, more than 40% admit they have fallen asleep at the wheel at least once while driving [6].

Fatigue can appear for many reasons. One significant cause of fatigue is the high level of traffic during peak hours of work access. This situation demands increased attention and concentration, which can lead to fatigue. Other reasons can be:

- Poor lighting, such as driving at night or early in the morning, also requires a higher level of attention and increases fatigue.
- The monotony of the environment (highways or dual carriageways).
- Haste requires greater concentration and can alter the psychophysical state.

In addition to these factors that enhance fatigue, the possibility of driving while already fatigued or drowsy must be included.

Accidents caused by drowsiness are up to twice as deadly as those caused by other causes [6]. In order to reduce these figures, strategies have been carried out, such as advertising campaigns, driving recorders in vehicles intended for the road transport of goods and passengers, or the use of drowsiness detection systems in automobiles. Within the scope of the latter area, the technologies used are diverse and can be based on the measurement of signals such as steering wheel movement, the position of the vehicle on the road, or driver monitoring [7]. Driver fatigue detection is a technology that has been exploited only a little, and that can be carried out with very different approaches [8].

In the automotive industry, various systems have been implemented to warn about the appearance of fatigue. These systems are known under different names, such as driver drowsiness detection (DDD) systems or driver drowsiness and attention warning (DDAW) systems [9]. Their presence until now was limited to packages of extras that are not included in the basic versions of the vehicles if the car model offers them. However, for new M and N category vehicle homologations, the EU requires the vehicle to implement DDAW systems [9].

DDD systems are usually grouped into three categories based on the type of variable being monitored [7,8]:

(1) Systems based on monitoring the behaviour of the vehicle.
(2) Systems based on monitoring the driver’s biomedical signals.
(3) Systems based on monitoring the physical characteristics of the driver.

Some of the advantages and disadvantages of each method are described below:

(1) The behaviour of the vehicle is understood in the sense that the car’s trajectory or speed can be disturbed by a careless attitude by the driver. This behaviour can be a great source of information. In this way, an idea of the level of attention can easily be obtained by evaluating signals such as the steering wheel’s speed or the angle of rotation. Drivers tend to pull off the road and make quick steering corrections when fatigued. Some manufacturers detect these corrections by monitoring, as mentioned above, the angle of the steering wheel and the steering behaviour [10,11].

The acquisition of these signals is simple, but it is important to note that it can be limited by road conditions. A bumpy or winding road makes this type of analysis difficult by introducing gait variations that are not actually disturbances attributable to driver drowsiness. These limitations underscore the urgency for better solutions in driver behaviour monitoring.

In addition, analysing the data and reaching a conclusion requires continuous monitoring and some time for sampling. Samples should be taken during a period of driving that is considered correct to establish a standard against which the rest of the journey can be compared. In this way, some characteristics are determined as deviations from the established pattern that are identified with erratic behaviour. This methodology may not be effective in situations in which the driver is drowsy from the beginning of the trip since it would not be possible to determine the reference driving pattern.

In addition, breakdowns or imperfections in the vehicle (in the pedal and steering wheel, vibrations, poor condition of ball joints, bearings, or steering rack) would also limit this type of analysis. Lastly, the system could mistakenly characterise the steering wheel turning pattern as drowsiness under adverse weather conditions, such as solid cross-winds.

Due to all these drawbacks, some manufacturers choose to monitor the vehicle’s position on the road with technologies that do not involve the mechanical elements of the car [12,13]. For instance, Ford chooses to recognise erratic driving by identifying the position of the vehicle relative to the lines of the road with frontal cameras [14]. Peugeot Societé Anonyme (PSA) does the same with a lane departure warning system using infrared (IR) sensors, justifying that they are cheaper and more robust in adverse weather conditions [15].

(2) Drowsiness detection systems are based on biomedical signals that measure the driver’s brain, muscular, or cardiovascular signals. Although it is true that systems capable of capturing biomedical signals non-invasively have been developed, in most cases, monitoring these signals requires the implementation of electrodes in the driver. This makes its commercial implementation easier if other ways of recording body signals without invasive elements are found [7,8]. 

The measurement of EEG signals is well-developed and can provide valuable data [16]. These signals are susceptible to movement, so their driving accuracy may not be good. If the need to implement electrodes in the driver is added to this, the viability of this system in the market is minimal.

Although the market is not ready to enter these products, they can be very useful at the laboratory level. The development of equipment for the detection of drowsiness requires validation processes with robust systems, such as the recording of EEG signals, which is a well-established and reliable method for analysing sleep in the health sector. In this way, EEG signals are often used as a reference to validate other drowsiness detection systems [16,17].

Regarding the analysis of ECG signals, the arrival of the smartwatch makes this category of systems regain some potential [18]. Until now, the systems present in commercial cars to monitor the signals of the driver’s heart are based on measuring the pulsations with equipment integrated into the steering wheel or in the driver’s seat. This has the disadvantage that the position of the driver or where he has his arms can prevent the measurement. However, wearing a non-invasive device, which means a device that does not require any penetration or incision of the skin, with an electrocardiogram, accelerometer and gyroscope function on the wrist can provide valuable opportunities for drowsiness detection by analysing Heart Rate Variability (HRV) signals and combining them with driver behaviour monitoring through other sensors available on a smartwatch [19,20].

(3) Drowsiness detection systems based on monitoring the driver’s physical characteristics use a camera or other types of sensors to analyse their behaviour. Artificial vision refers to visual analysis using image processing techniques, as in systems based on driver behaviour analysis; artificial vision’s fundamental advantage is zero intrusion on the driver’s body. Using cameras located inside the vehicle’s passenger compartment, facial expressions, eye activity, yawning, or nodding, among other behaviours of the driver, can be monitored [21,22,23]. 

The technical proposals to detect drowsiness under the principle of artificial vision are very varied. Different types of cameras (IR, non-IR, stereoscopic), different algorithms [24,25], and different classification and image recognition engines are proposed in the literature [26,27,28].

The main handicap of this type of technology is the conditions in which the image is taken. Light conditions or accessories such as glasses or hats can make it challenging to analyse the image, which already has a high computational cost.

Different scales have been proposed to measure drowsiness subjectively, i.e., the subject assesses his or her level of drowsiness. These scales are carried out using questionnaires or interviews in which the driver describes his or her state of drowsiness [7]. The best-known and regarded benchmark is the Karolinska Sleepiness Scale (KSS) [29], developed at the Karolinska Institute of Sleep Medicine in Stockholm, Sweden. The KSS is a nine-point scale ranging from “extremely alert” to “very sleepy, great effort to stay awake, struggling with sleep”. Since the questionnaire data requires time to be completed, this method is unsuitable for real-time drowsiness detection. However, KSS has been used as a benchmark to check the accuracy of other systems [30].

Some research has focused on the simultaneous combination of several of the previous approaches [31,32,33,34]. These methods combine the advantages of different approaches to detect driver drowsiness (Table 1).

Generally, approved systems to control drowsiness are limited to generating sleep alerts to recommend breaks to the driver [10]. In some cases, there are procedures that the driver can activate, giving the vehicle some autonomy to carry out corrective actions, such as adjusting the steering wheel to keep the car within the lane or slowing down until the vehicle stops [35]. 

Considering the previous arguments, this work addresses the recognition of the physical characteristics of the driver that show signs of drowsiness using a multidimensional index. This composite index takes into account various physical characteristics, such as the frequency of blinking, yawning, and eye-opening, as well as the gaze direction and head position. The goal is to determine the state of the drivers by monitoring these facial expressions. For this purpose, an algorithm capable of detecting drowsiness has been developed. Two different approaches are used: Facial recognition based on Haar features and facial recognition based on Histograms of Oriented Gradients (HOG). The implementation has been carried out on a Raspberry Pi, a low-cost device that allows the creation of a prototype that can detect drowsiness and interact with peripherals such as cameras or speakers.

The novel contribution of this paper is the proposal of a five-index method to detect drowsiness while driving. The detection algorithm includes, in addition to the usual EAR and MAR indices, the calculation of PERCLOSE, gaze direction and head orientation. This multifactorial approach results in a higher performance, fewer false alarms, and more robustness to drowsiness detection, as shown in the Section 3. The proposed multi-index methodology is valuable compared to one-dimensional algorithms focusing on one-index detection, such as Eye Aspect Ratio (EAR). There are different situations in which eye detection becomes more confusing or complicated. A person may close or squint their eyes for a variety of reasons, for example, because they are yawning, want to focus on a distant object, or are in direct sunlight. In the latter case, most people wear sunglasses, whose opacity prevents the eyes from being monitored. This research also aims at the experimental determination of personalised thresholds for each of these five indices and for different drivers. These thresholds are shown in the Section 3.

The advantages of the developed method and its implementation in a low-cost device are that it allows detecting drowsiness under actual driving conditions, with real-time analysis, and with the possibility of being installed in any vehicle.

The rest of the paper shows the procedure carried out to develop the proposed methodology. In Section 2, the materials and the algorithms developed for the detection of drowsiness through facial recognition are presented. In Section 3, tests are carried out to evaluate the validity and usefulness of the developed method. In Section 4, the results obtained are discussed, and the advantages and limitations of the solution adopted in this work are highlighted. Finally, some relevant conclusions are drawn in Section 5.

## 2. Materials and Methods

### 2.1. Materials

This subsection describes the materials used in this research to implement the drowsy driving detection prototype.

Raspberry Pi 4 Model B has been used. It has a 64-bit quad-core processor running at 1.5 GHz. The Raspberry Pi is a low-cost single-board computer known for its versatility. At the development level, the Raspberry Pi 4 offers many features that simplify the work. It has HDMI image outputs, which are perfect for connecting the board to any monitor. It also has Wi-Fi connectivity, which is very useful for downloading all the tools required for this work. In addition, it has a dedicated port to connect a camera.

As for the power supply, it works at 5.1 V, so to install it in the car, it has been necessary to use a charger that connects to the 12 V socket and converts it to 5.1 V. According to Raspberry’s official data, the current it draws is around 600 mA, and it can reach 1.25 A under maximum load. This is a maximum power consumption of 6.25 W, but due to the use of peripherals, a power supply that can deliver up to 3 A (15 W at 5 V) has been used.

The camera is a fundamental element for drowsiness detection based on facial recognition. A camera is required to obtain images in different lighting conditions, both day and night, when driving outside and in a tunnel. Any camera will take images in good light conditions, but a specialised camera is needed to capture the environment without light.

Working with the Raspberry Pi has the advantage of having its official camera for IR photography. This way, accompanied by an IR lamp, the face can be captured without visible light. The camera used in this research is the Raspberry Pi NoIR Camera v2, whose IR filter (usually built into the lens by default) has been removed.

With the selected camera, the image taken has reddish tones when light is present. This is not a problem as colour is not essential for facial recognition, and the image is converted to greyscale. When there is no light, the camera does not manage to capture anything. Therefore, an IR LED lamp has also been installed in the passenger compartment so that when there is no visible light, the camera can take images thanks to the presence of IR light without a filter that blocks these waves.

The selected IR LED lamp is a cost-effective 24 LED board. The choice of an 850 nm wavelength model is deliberate. IR lamps with a wavelength close to the visible spectrum emit a faint red glow when in operation, providing a quick and visual confirmation of the lamp’s functionality. This eliminates the need to use the camera for verification. Once the prototype is validated, an upgrade to a 940 nm lamp, which emits no perceptible glow, can be considered without compromising the camera’s image capture capabilities.

Another key feature of the lamp that influenced the decision is its power supply voltage, which is a standard 12 V in DC, commonly found in car power supplies. This compatibility makes the lamp easy to install, as it can be directly connected to the car’s 12 V socket or to any equipment powered by this voltage, such as light bulbs or radio.

An exciting feature of the lamp is the presence of a light-dependent resistor (LDR) that regulates the operation of the LEDs, turning them off when there is light and turning them on when there is not.

### 2.2. Methods

This section describes the methodology used in this work to detect drowsiness. The different steps of the detection method are presented under the following headings.

#### 2.2.1. Face Detection and Coordinates Extraction Methods

First, the camera continuously monitors the driver’s face. In bright conditions, the captured image shows reddish tones due to using a camera without an IR filter. This does not interfere in any way with the performance of the system. It does not affect the face detection and coordinate extraction processes. In dark conditions, an IR lamp provides the necessary light for the camera to extract information from the scene. The resulting images show almost no chromaticity, which does not interfere with the system as the algorithms themselves convert the images taken to greyscale to work with them.

For the detection of the face in this work, two classifiers have been compared: the Haar cascades method [36] and the Histograms of Oriented Gradients (HOG) method [37,38].

The Haar cascades method, based on cascade classifiers using Haar functions, is a highly efficient method for facial recognition tasks. The algorithm, as described by Paul Viola and Jones in [36], has a low computational cost, making it suitable for real-time applications.

In this work, Haar cascade recognition is conducted with the OpenCV (Open Computer Vision) library [39], a computer vision library developed by Intel. It is the most popular artificial vision library and allows the system to carry out tasks such as motion detection and shape recognition, with facial recognition being one of its essential applications. It is cross-platform, and it works on both Windows and Raspberry OS. It is free (under a license that allows it to be used freely for commercial and research purposes), and it has complete documentation on its website, so it fits perfectly in this work. It is developed in C++, but it includes connectors for several languages, such as Python.

HOG detection is based on the idea that the appearance and shape of a face can often be characterised by a gradient distribution of intensities and edge directions [40,41]. Most of the time, it is more important to analyse the shapes than the colour to detect objects. Therefore, like the detection with Haar classifiers, this method works with grayscale images, so the intensity gradients refer to luminosity.

The HOG method transforms the amount of information available in the image into a smaller volume that provides the important data. The obtained vector does not provide visual information with which the image can be seen through its data, but these are very valuable when combined with a Support Vector Machine (SVM) image classifier [42]. When training a classifier with the SVM algorithm, a set of positive images (of faces) and a set of negative images (that do not contain faces) are used, and what is performed is to process the extracted feature vectors according to the exposed method. The classifier can be trained with faces from the front, in profile, from above, etc. In the case of facial detection in driving, according to the strategy that is being considered, it is enough to be able to recognise a face frontally. In this work, HOG recognition is implemented using Dlib, a powerful and free C++ library [43].

**Figure 1:**
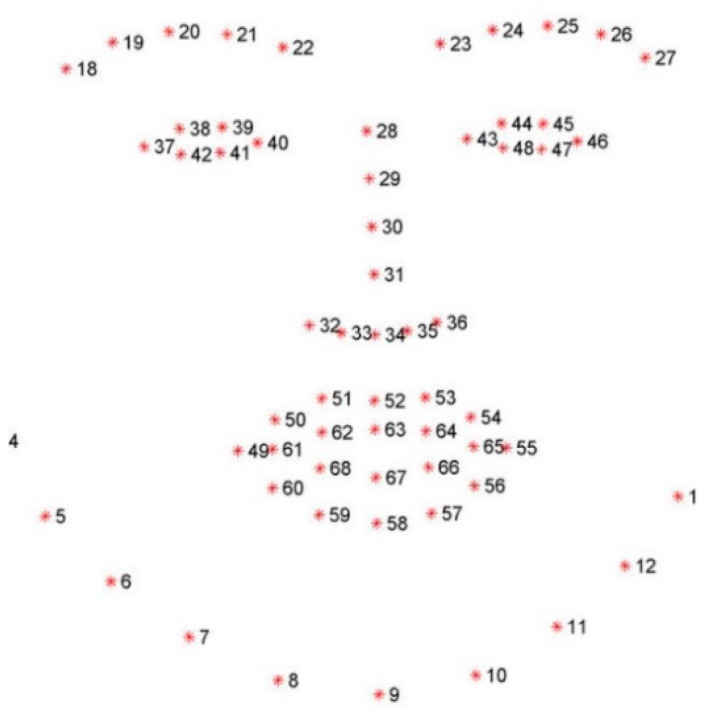
Dlib facial coordinates.

In addition to facial detection using HOG, the Dlib library contains an essential function for the extraction of facial coordinates (Figure 1).

As can be seen in the coordinate map that Dlib works with, each eye is defined by a set of six coordinates and the mouth is represented by a set of twenty points.

#### 2.2.2. Calculation of Drowsiness Indicators

This section presents the five indices used in this work for the detection of drowsiness.

##### Yawn Indicator

Yawning is an involuntary act consisting of opening the mouth and reaching a wide separation of the jaws. The excessive opening and an average duration of approximately seven seconds make it an easily recognisable expression. In addition, it is accompanied by complementary gestures: when yawning, it is expected to tilt the head back and squint the eyes. Additionally, many other events occur but must be recognisable by visual analysis.

A yawning study methodology could be based on the degree and time of mouth opening. Although not all humans have the same features, it is easy to differentiate a yawn (mouth wide open) from a closed mouth. Therefore, by morphologically analysing the region of the mouth through image recognition, the geometry can be extracted at each moment, and a yawn can be detected [44].

**Figure 2:**
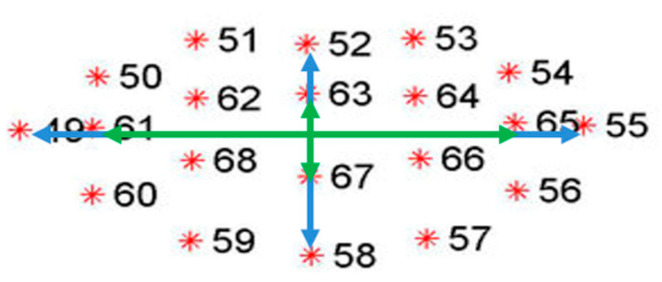
Mouth coordinates.

One of the most widely used indicators to discern between an open mouth and a closed one is the Mouth Aspect Ratio (MAR) parameter [44,45]. This parameter is a ratio between the length and width of the mouth (Figure 2).

Not all authors use the same formula to assess the degree of mouth opening [44]. As Figure 2 shows, the length and width of the mouth can be defined with the inner or outer coordinates of the lips. In addition, there are several pairs of coordinates facing each other vertically, and there is no combination of points that is indisputably more significant.

**Figure 3:**
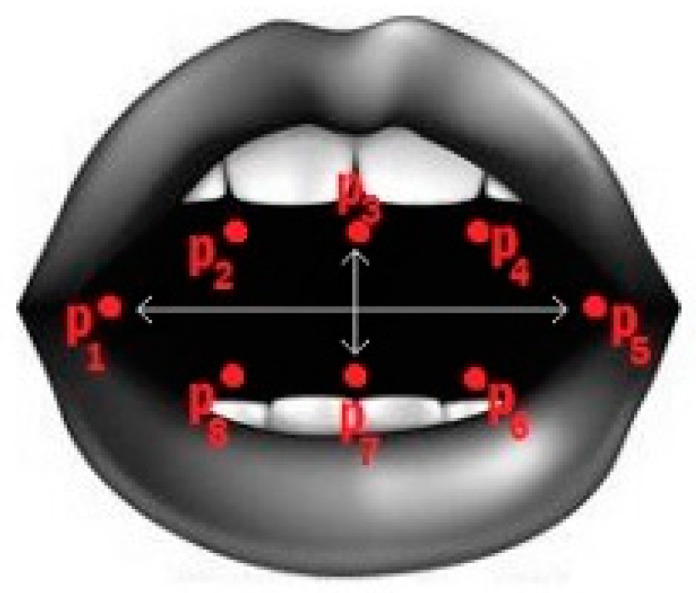
Points are used to determine the degree of opening of the mouth.

Thus, in the literature, the MAR parameter is found to be calculated both with interior and exterior coordinates of the lips and with pairs of coordinates according to the author’s criteria. For this work, the points that define the inner contour of the lips will be used. This criterion has been chosen because, with this configuration, a complete contour of fewer points than the outer contour is taken (Figure 3).

If it is possible to detect the mouth in an image and extract the indicated coordinates, its opening can be evaluated with Equation (1).

(1) $$MAR=\frac{{|p}_{2}-p_{8}\left|+{|p}_{3}-p_{7}\right|+{|p}_{4}-p_{5}|}{2\cdot {|p}_{1}-p_{5}|}$$

##### Eyes Opening Degree Indicator

Vision can become blurred under the effects of fatigue, causing a decrease in visual acuity. In extreme cases of fatigue, there are even optical illusions, such as the perception of brightness, shadows, or deformations of the environment. These effects obviously limit the abilities involved in driving, but this work must be focused on the signs of the eyes that reveal drowsiness.

The frequency of the blinks increases considerably with drowsiness, as well as the time they remain closed in each blink. This causes the blink period to decrease as well as the percentage of this period during which the eyes are open.

In addition, the eyes often remain partially closed in conditions of fatigue. Anyone who has experienced drowsiness, not necessarily while driving, has felt the heaviness of the eyelids, how the eyes close and how it takes effort to keep them open.

**Figure 4:**
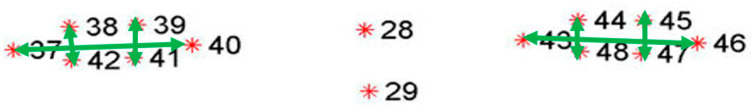
Eyes coordinates.

For the eyes, the most widespread study indicator is the Eye Aspect Ratio (EAR) [46]. Its foundation is the same as that of the MAR, and therefore, the analysis procedure is similar. A six-point contour of each eye is obtained to extract the coordinates of the eyes with Dlib, as shown in Figure 4.

**Figure 5:**
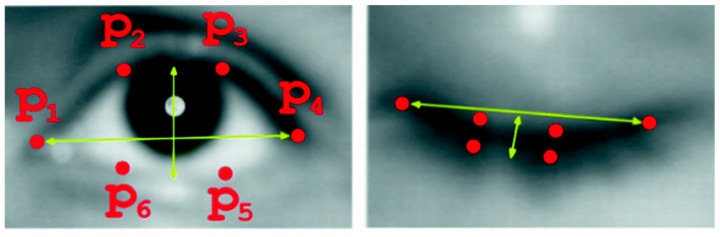
Representation of EAR coordinates for open and closed eyes.

Unlike the MAR, the publications that use the EAR parameter converge towards the same solution [47,48,49]. As there is a single contour formed by only six points, this indicator is calculated according to Equation (2) using the point map in Figure 5.

(2) $$EAR=\frac{{|p}_{2}-p_{6}\left|+{|p}_{3}-p_{5}\right|}{2\cdot {|p}_{1}-p_{4}|}$$

##### Percentage of Time with Eyes Closed Indicator (PERCLOS)

The PERCLOS parameter is used to obtain the percentage of time the eyes remain closed. This term refers to the acronym PERcentage of the time eyelids are CLOSed. It is an index that calculates the percentage of time the eyes remain closed below a certain reference level of aperture. It is one of the best-known criteria applied in the literature to detect fatigue or drowsiness in drivers, for example, in various studies carried out by the National Highway Traffic Safety Administration (NHTSA) [12].

It is calculated by dividing the time the eyelid is closed below the set threshold by the total time period observed (Equation (3)). It is then multiplied by 100 to obtain the result as a percentage.

(3) $$PERCLOS=\frac{eyelid\;closure\;time}{total\;time\;lapse}\cdot 100$$

A high PERCLOS value indicates a higher percentage of time the eyes are closed and this is associated with drowsiness or fatigue, suggesting that the subject is in a reduced state of alertness.

##### Head Position and Tilt Indicator

To estimate the head position, the three Euler angles are calculated: pitch, roll and yaw. These three angles describe the three-dimensional orientation of an object relative to a fixed reference.

Using a 3D model of a generic human head and the position of the facial landmarks obtained with the landmark predictor, the rotation and translation of the head are calculated.

##### Gaze Direction Indicator

The Gaze Score indicator is derived from the euclidean distance between the centre point of the eye and the centre point of the pupil. In this way, it is possible to determine whether the driver is also looking straight ahead when his head is facing forward or whether his gaze is distracted.

The region of interest of the eye is already defined by the key points of the predictor, and the Hough transform (Equation (4)) is used to identify the pupil and its centre.

(4) $$Gaze\;Score=\frac{distance\;L2}{eye\;width\;}$$

where L2 distance is the euclidean distance between the centre point of the eye and the centre point of the pupil and the eye width is the distance between the extreme ends of the eye.

##### Assessment of Drowsiness Indicators

**Figure 6:**
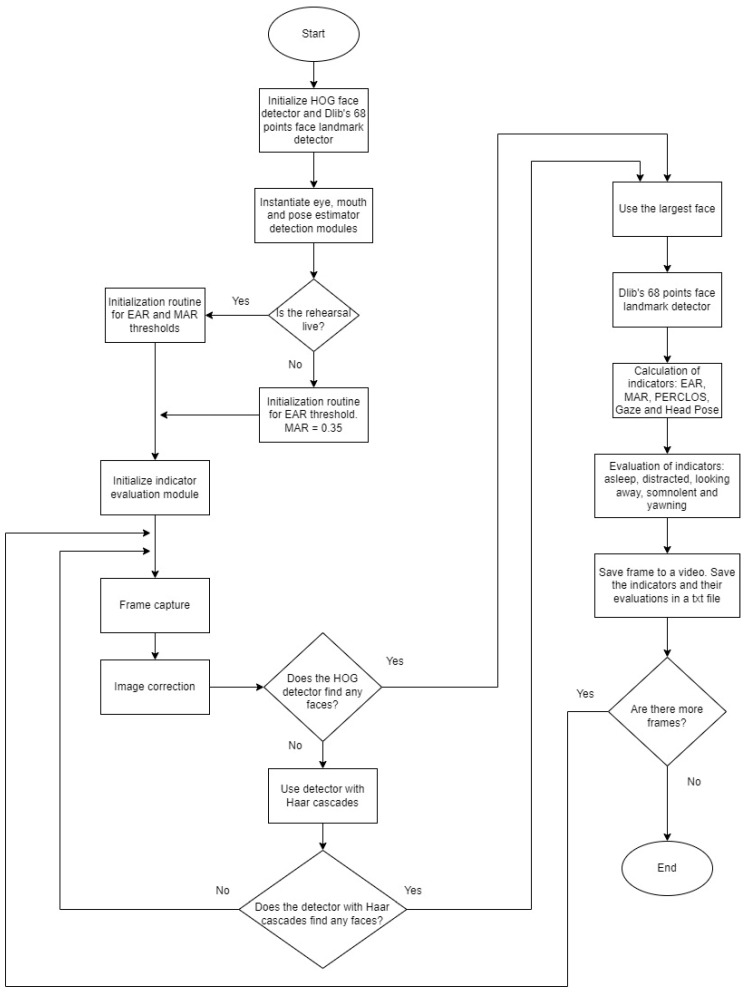
Flowchart of the drowsiness detection algorithm.

This section presents the algorithm developed for the detection of drowsiness. The main objective of the algorithm is to classify the five indicators presented in the previous section and generate five different alarms that are not mutually exclusive: asleep, distracted, distracted gaze, drowsy and yawning. Figure 6 presents the flowchart of the developed algorithm. The thresholds used in this work for each indicator, based on the performed tests, are presented in Table 2.

The definition of the thresholds is a key part of the development of this system, as exceeding them will depend on whether or not a drowsiness condition is detected in the driver. The thresholds corresponding to the EAR and MAR indices, i.e., EAR_thresh and MAR_thresh, are defined in the algorithm’s initialisation routine. The main reason is that eye and mouth openings are very person-dependent features, and depending on their features, they will have different thresholds. Priority is given to EAR_thresh because it has the most variability. The MAR_thresh can be defined by the initialisation routine if the test subject keeps his mouth open for the duration of this routine. If this is not possible, as in the case of dataset testing, then an average threshold value of 0.35 is considered.

It should be noted that the parameters referring to the direction of the head and gaze are considered to be centred when the subject is facing the camera. This was decided because face detectors also obtained better results, with the face as frontal as possible. For this reason, the priority when mounting the camera in the vehicle is to position the camera as centrally as possible to the driver.

The starting point is face detection. As discussed above, the pre-trained Dlib model called “get_frontal_face_detector”, based on the HOG method, was used to detect the face. The Dlib detector performs well but has limitations when detecting faces that are not in a relatively frontal position. A combination of HOG and the Haar cascades method has been developed to overcome this. The priority detector used has been HOG because it is the best-performing algorithm in the tests carried out, but if it fails to detect a face, the Haar cascade classifier is used.

The capture of the input image is executed with the OpenCV library, which captures images in BGR format, meaning that the order of the colour bands is represented in that order: blue, green and red. In order for face detection to be successful, the detection algorithm is given an image with the highest possible quality, and image pre-processing techniques are used for this purpose. The following have been used:

- Image conversion in greyscale BGR format: the image taken by the camera is represented in a three-channel colour model; however, for the chosen detector, the image is required to be in one channel only, i.e., greyscale.
- Bilateral filter: this is a non-linear filter that preserves detail and reduces noise in images.

The image obtained after image pre-processing is used as input to the face detector. The output of this detector is a vector whose content is the coordinates of the rectangles with a face inside them. In this case, relevance will only be given to the most prominent face found, which is assumed to be the driver’s. In this way, the area of the image in which the elements of a face have to be searched for and located is delimited. This task is carried out by the predictor “shape_predictor_68_landmarks”, which, as already mentioned in this paper, is the predictor of the 68 key facial points of Dlib. The output of this predictor is a vector, called “landmarks”, with the positions of the detected facial points on the driver’s face.

From this vector, the eyes and mouth are located, and the contours of both are represented in the image. Then, this same vector is introduced in the module calls: Eye_Mouth_Detector_Module to obtain the EAR, MAR, and Gaze indicators; Pose_Estimation_Module to obtain the roll, pitch, and yaw values; Atten-tion_Scorer_Module to calculate the PERCLOS and evaluate the rest of the indicators. The scores of the indicators are displayed in the analysed frame, which is then saved in the video. Saving the frames in the video is not necessary once the efficacy is tested, but in the trials, it is relevant to have the video available and to be able to play it back later for testing purposes.

Finally, the results obtained in each frame are written in a text file and then analysed.

The Attention_Scorer_Module performs the evaluation of the parameters calculated in the previous module. It also calculates the PERCLOS index from the EAR index. The subject is evaluated in such a way that five different alarms can be given: asleep, distracted, gaze distracted, yawning and drowsy. These can be given simultaneously.

- The snooze alarm is related to eye openness with the EAR index. When a subject’s eyes remain closed below the set threshold for a set time, e.g., below 75% of aperture and for 3 s, a sleep alarm is generated.
- The inattentive alarm is obtained from the subject’s pose by observing the position of the subject’s head. If any of the Euler angles (roll, pitch and yaw) that are calculated to estimate the head position are above the set threshold for a certain time, the subject is considered not to have his or her head facing forward, and the inattentive alarm is generated.
- The distracted gaze alarm refers to when the subject has their head positioned towards the front, but the direction of their gaze is not centred. The gaze score is used for this purpose, and when the gaze score is above the established threshold, the gaze distraction alarm is generated.
- The yawning alarm occurs when the mouth opening is above a certain threshold for a specific time. For example, if the mouth is open more than 50% for 3 consecutive seconds.
- The tired alarm, which in this case is used as drowsy, refers to the amount of time the subject spends with eyes closed below a certain threshold, i.e., the PERCLOS index. For example, if the subject spends more than 20% of the test time with eyes closed below this threshold, the drowsy alarm is generated.

Regarding the calculation of PERCLOS, in this work, the calculation is made every minute. The time in which the eyes are closed below the established threshold is accumulated, and then this data is divided by 60 s. In this way, it is possible to observe the PERCLOS during each minute, but it is also possible to calculate the global PERCLOS by averaging the scores obtained at the end of each minute, and it is also possible to detect the moments in which there have been episodes of more occlusions by analysing the record of the path.

**Figure 7:**
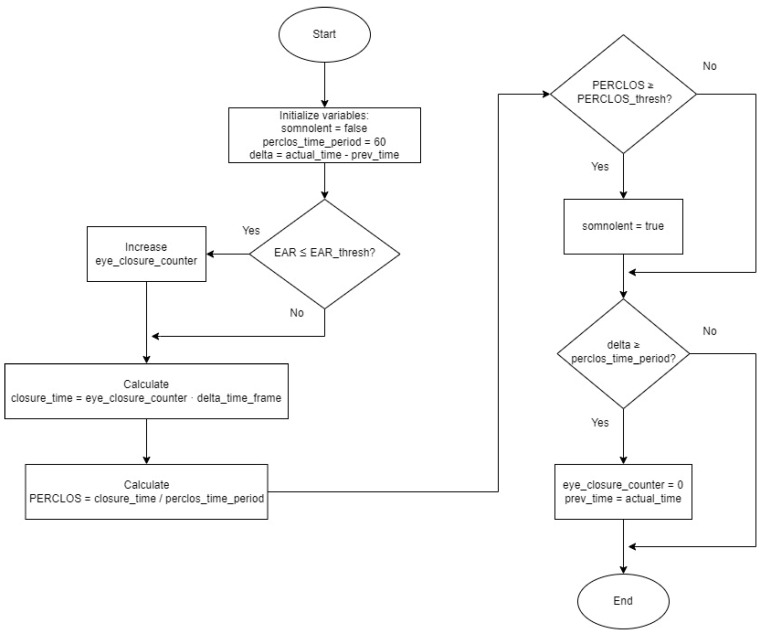
Flowchart of the PERCLOS evaluation function.

Figure 7 shows the flowchart of the function that calculates PERCLOS. It should be noted that the following global variables have been defined prior to this function:

- prev_time = 0 is an auxiliary variable to calculate the time that has elapsed.
- eye_closure_counter = 0 is the counter of the number of frames in which the eyes have been closed, i.e., the EAR parameter has been less than its threshold, the EAR_thresh.
- delta_time_frame = 1/capture_fps is the estimated time that a frame lasts, where capture_fps is the frames per second of frame capture.
- perclose_time_period = 60, is the time in seconds defined to calculate the PERCLOS, in this case, 60 s.

In addition, an initialisation routine was performed on the subject who would behave as a driver in the vehicle. The reason for this routine is that each person has different physiological characteristics, so a customised calculation of the EAR and MAR in-dices has been incorporated to monitor the opening of the eyes and mouth as accurately as possible. In the tests, the subject is asked to keep their mouth as open as possible for the first 5 s. In this way, an estimate of the subject’s mouth-opening ability can be obtained.

The EAR_thresh threshold is defined as three-quarters of the average EAR during the initialisation seconds (Equation (5)).

(5) $$EAR_{thresh}=EAR_{mean}\cdot 3/4$$

It is therefore defined that if the eyes are closed below 75% of their opening capacity, they are too closed. If the score is below this threshold during the time stipulated for the eyes to be closed, it will be related to the states of sleepiness and drowsiness.

The MAR_thresh threshold is calculated as half the average MAR over the seconds of the initialisation routine (Equation (6)).

(6) $$MAR_{thresh}=MAR_{mean}/2$$

It is thus decided that when the mouth is open to more than 50% of its opening capacity, it is sufficiently open to be considered a yawn if the yawning time is exceeded.

In this work, once drowsiness is detected, no action is taken on the vehicle. For the development of the system, visual alarms have been chosen, displayed on the screen, and stored in the video that is captured during the test so that it can be analysed later. The final system must have an acoustic alarm that alerts the driver when, due to their fatigue, they are not paying attention to the vehicle’s dashboard. This will be accompanied by a sign on the vehicle dashboard informing the driver of the need to take a break and the vibration of the steering wheel, where applicable.

## 3. Results

This section analyses the results of the indicators used in the algorithm throughout the different types of tests and trials. Tests have been carried out on different subjects, with different accessories, and with variability of lighting conditions.

**Figure 8:**
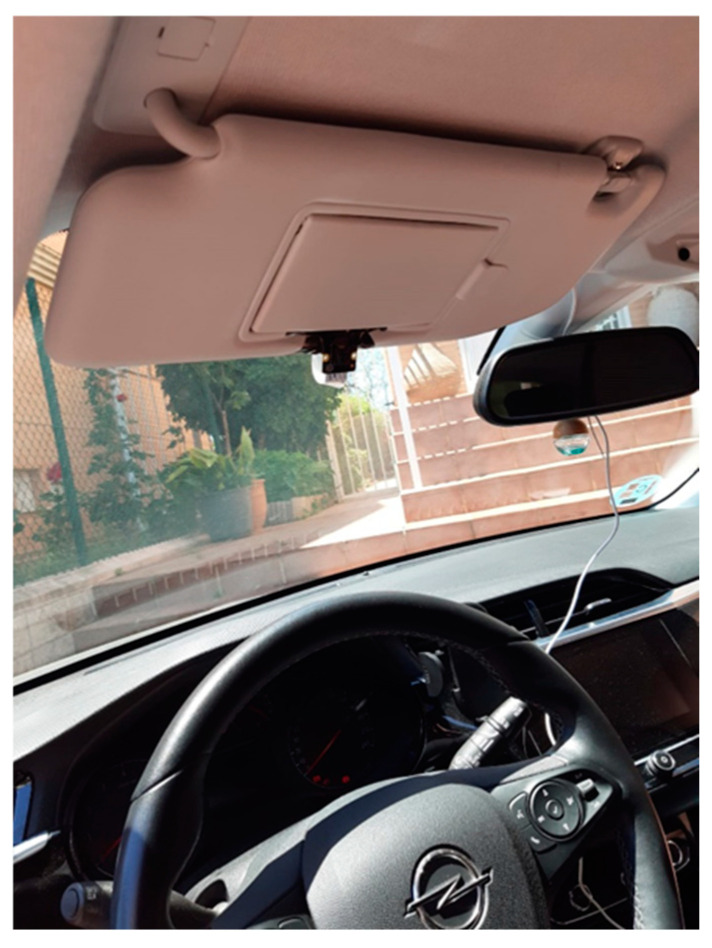
Camera assembly in the car.

The installation of the camera in the vehicle is one of the critical points since the face detectors’ characteristics mean that priority must be given to placing the camera as frontal to the driver as possible. The system has been installed in an Opel Corsa 1.2T XHL Elegance vehicle. After testing different placement options for the system, such as on the dashboard, it was decided to place it on the sun visor of the driver’s seat, ensuring that the camera is aimed correctly at the driver. In this way, the sun visor does not obstruct the driver’s field of view and, in turn, allows the camera to be at the subject’s eye level, considerably improving the algorithm’s results. Figure 8 shows the camera setup.

**Figure 9:**
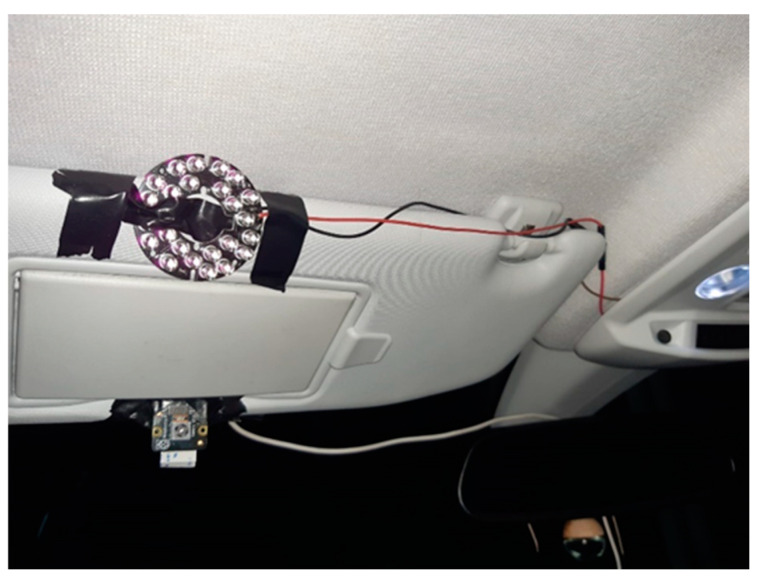
Position of the infrared LED lamp in the car.

For tests in low light conditions, such as at night, the infrared LED lamp was mounted by making the connections to one of the 12V courtesy lights at the front of the vehicle (Figure 9). It should be noted that the LDR has been removed from this lamp in order to regulate the LEDs according to the ambient lighting, as there were problems with street lamps or elements that provide light during nighttime tests, which caused the LEDs not to light up. To circumvent this problem, it has been decided to short-circuit the LDR so that the LEDs remain on for as long as they are powered.

A total of eleven participants took part in the tests. The experimental subjects were informed of the scope and objectives of the tests and signed an informed consent form. Each subject completed one or two sessions lasting 20 min each. The gender of the subjects is 64% male and 36% female. The age range is between 23 and 58 years. Each participant had specific circumstances that were considered to influence drowsiness, and these were reflected in the form they filled in prior to the tests. The results of all the tests conducted are included in Appendix A.

The tests have been carried out in different driving scenarios and with different routes to give more variability in this aspect, some of them in town and others on the motorway. The conversation between the participant and the accompanying person is restricted to only the response to the subjective estimation of the KSS index, which is carried out every 10 min. In this case, at the start of the test and right in the middle of the test.

**Figure 10:**
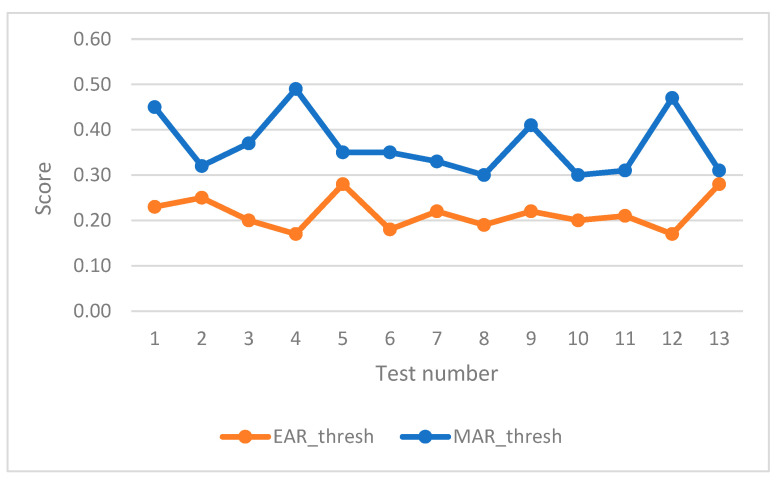
Variation of users customised thresholds in the tests.

The first few seconds of the test are used for the initialisation routine of the driver’s face, which establishes the thresholds for the EAR and MAR indicators. The customisation of the MAR indicator threshold has made it possible to adjust the mouth opening reference and, consequently, the occurrence of yawning alarms for each subject. Figure 10 shows the different results of the thresholds obtained throughout the tests carried out.

**Figure 11:**
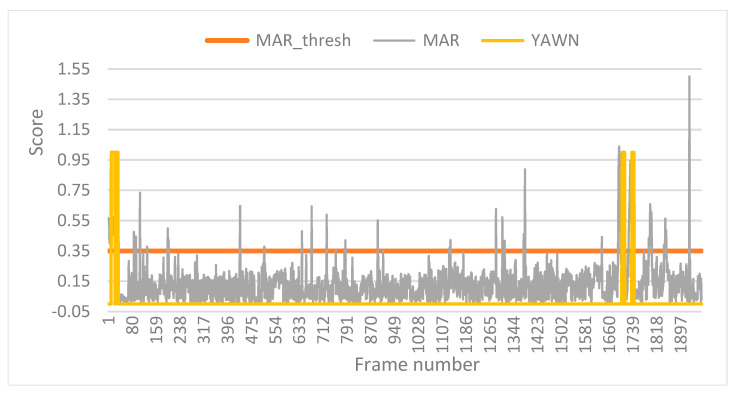
Evolution of the MAR in test 6.

As an example, Trial 6 is shown in Figure 11, in which the MAR indicator, the MAR threshold, and the yawn alarms (Yawn) are plotted together. On three occasions, the yawn alarm occurred because the MAR score exceeded its threshold during the time considered a yawn.

**Figure 12:**
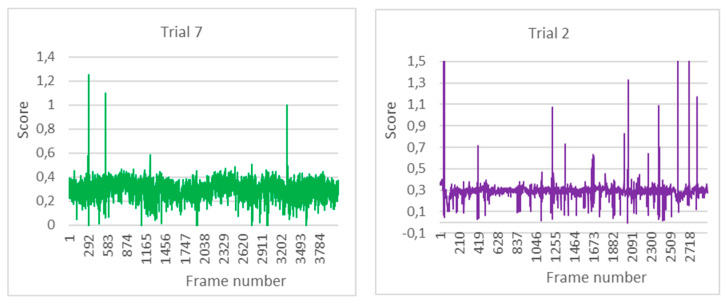
Evolution of the EAR in tests 7 and 2, respectively.

The customisation of the EAR threshold has been decisive in adapting the eye-opening reference to each subject. As can be seen in Figure 12, there was some variability in the results of the personalised thresholds obtained. Moreover, even in the same subject, different scores were obtained, probably due to the combination of the time of the test and the subject’s condition at the time.

In the first graph in Figure 12, corresponding to test 7, the variation of the EAR indicator is between 0.1 and 0.4. There are some points where the eyes were either much more open, exceeding the unit score, or much more closed, approaching zero. The EAR_threshold for this trial was 0.22, which, although below the EAR score for this trial on some occasions, did not last long enough to trigger the sleep alarm.

In the second graph in Figure 12, the vertical axis has been adjusted to the maximum EAR score of 1.5 to better appreciate the variation in most of the tests. There are only three values that exceed this score and are considered isolated. In this trial, number 2, there has been less variation around the average score, which is above 0.3, above its EAR_threshold of 0.25.

The EAR indicator has been used to activate the snooze alarm. If the EAR score is below its threshold, the EAR_threshold, for the time stipulated in the parameters, the snooze alarm occurs.

**Figure 13:**
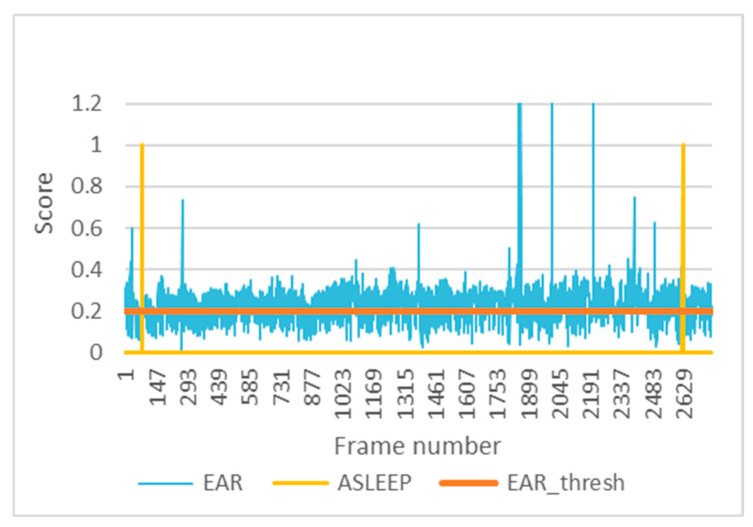
EAR and snoozing alarm in test 1.

Figure 13 shows the evolution of the EAR in test 1 under driving conditions together with the EAR_threshold, of value approximately 0.2, and the activations of the sleep alarm. These alarms occur just after three consecutive seconds in which the eyes are closed below the threshold.

**Figure 14:**
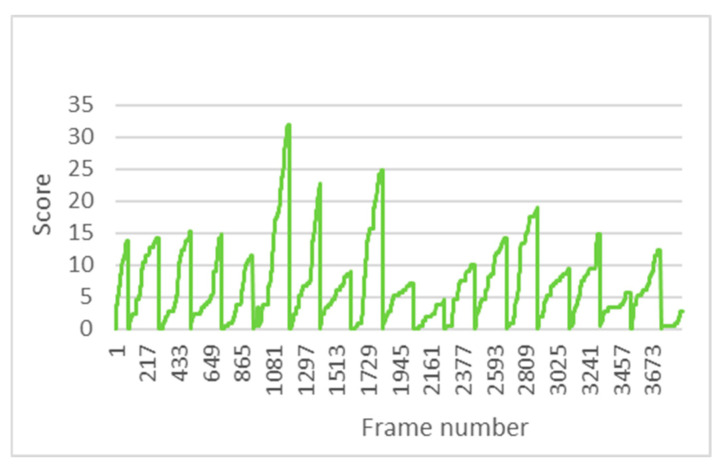
Evolution of PERCLOS in test 11.

The PERCLOS indicator makes it possible to assess the driver’s drowsiness state through the amount of time the eyes remain closed, so the EAR threshold is crucial for a good PERCLOS calculation. Having customised these thresholds, PERCLOS is thus adjusted to the physical characteristics of the individual driver. Figure 14 shows the evolution of PERCLOS in one of the driving tests, where each minute shows a cumulative score for this indicator. At minute 6, the maximum score is around 32%.

**Figure 15:**
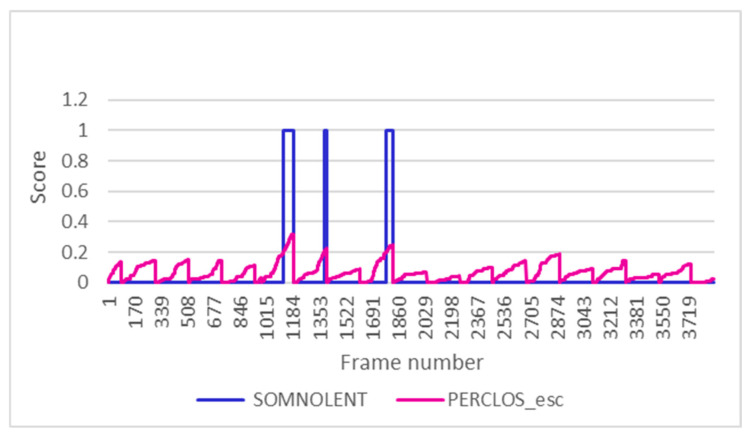
Activation of drowsiness alarm in test 11.

As mentioned in the previous section, this indicator is used to determine whether or not the drowsiness alarm is triggered. In order to represent the activation of this alarm together with the PERCLOS in a graph and to be able to visualise it correctly, the PERCLOS has been scaled between 0 and 1 (PERCLOS_esc). Figure 15 shows how the drowsiness alarm is triggered when the PERCLOS score exceeds 20%. This turns out to be in three different minutes, and in the rest, the alarm remains deactivated.

**Figure 16:**
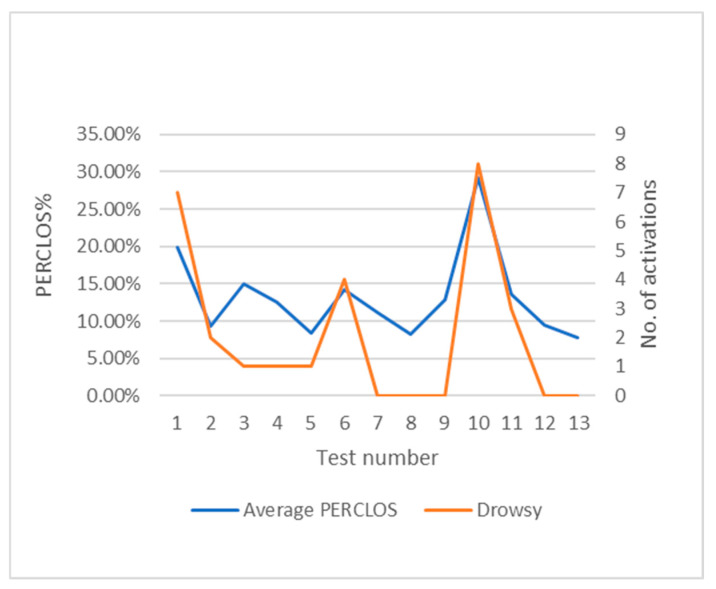
Average PERCLOS score and drowsiness alarm across tests.

The PERCLOS score has been averaged using the cumulative scores for each minute of testing. Figure 16 shows the different average PERCLOS scores obtained throughout the tests under driving conditions.

**Figure 17:**
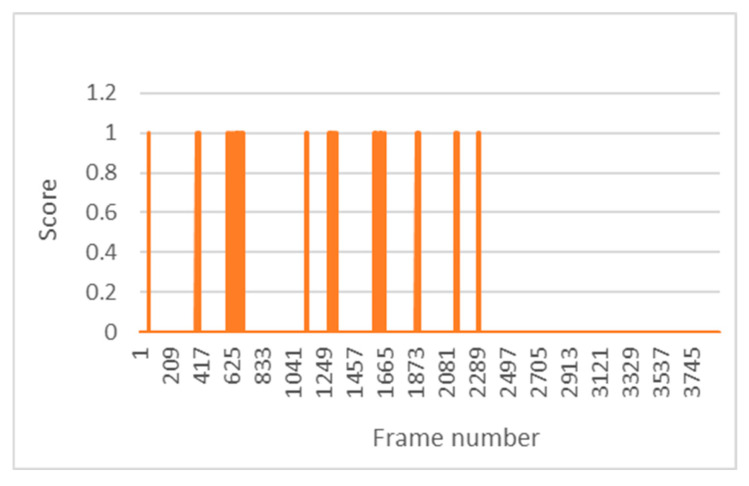
Distracted alarm activations in test 8.

Euler angles were used to recognise the position of the head. From these angles, a usual range of movement has been defined in which the driver is considered to be focused on the road. Outside this range and for more than six consecutive seconds, the driver is considered to be distracted, a situation that triggers the distracted alarm. Figure 17 shows the activations of the distracted alarm based on the Euler angles to locate the driver’s head. This is test number 8, with nine alarm activations recorded.

However, it should be noted that the calculation of Euler angles has sometimes been limited by the face detector, which performs best when the head is in front of the camera. For this reason, and because driving is full of stimuli that are sometimes difficult to determine, these alarms have been considered among the least relevant.

Finally, the gaze indicator was used to trigger the gaze distraction alarm in order to establish a difference between moving the head and changing the direction of the gaze.

**Figure 18:**
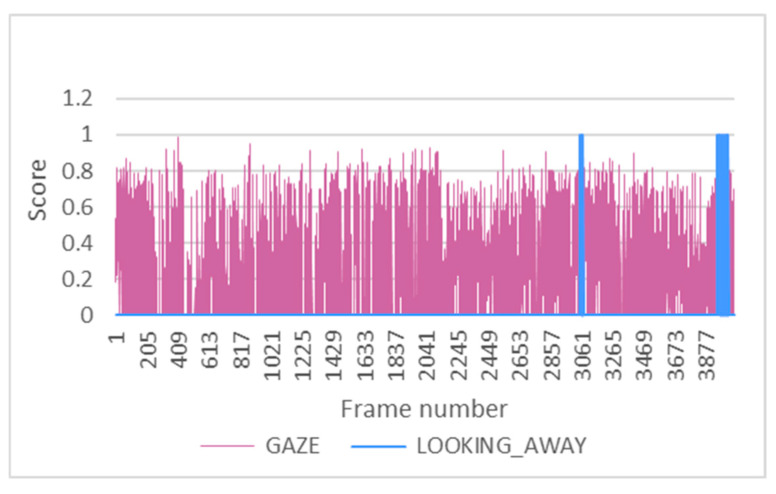
Gaze and gaze distraction alarm in test 7.

In most tests, no gaze distraction has occurred, as it is required that the face detector correctly locates the driver’s face, the eyes are located, and the iris is sufficiently well differentiated from the sclera of the eye. In addition, a deviation of the gaze for at least four consecutive seconds is required to trigger the alarm. Figure 18, from test 7 under driving conditions, shows two activations of the gaze deviation alarm. It can be seen how the activation occurs after several frames, corresponding to more than four consecutive seconds, exceeding the Gaze_threshold. The value of this threshold has been previously defined in the algorithm with a value of 0.4. It corresponds to the excessive deviation of the gaze, which means it is not centred.

## 4. Discussion

Throughout all tests, face detection rates have been found to be high, with positive face detection rates above 95% in the vast majority of cases. The HOG detector is much more effective, as was assumed from the beginning of this work. The decision to include Haar cascades as a secondary detector was an attempt to cover times when the HOG detector was not sufficient.

In the analysis of the EAR and the MAR, a processing speed of between 3 and 4 fps has been obtained. This processing speed allows adequate operation of the drowsiness detection algorithm given the duration of the physiological phenomena to be detected. The squinting of the eyes in the case of the EAR is constant over time in the presence of drowsiness, so having a lower sampling frequency does not affect the result. This fact would be different if it were a matter of detecting a phenomenon of much shorter duration. In that case, the sampling frequency would have to be increased due to the risk of losing key information.

The same can be said for MAR and yawn detection. The average duration of a yawn is between 4 and 7 s, so, in the same way, it can be considered a phenomenon long enough in time so that its detection does not require a higher sampling frequency.

It should be noted that the processing speed obtained by the algorithm executed on a commercial laptop reached 15 fps. The implementation of the algorithm in dedicated electronics would increase the processing speed.

It is worth noting that the algorithm’s performance improves when driving in environments with few distractions and mostly straight roads rather than in crowded ones because, in the former, there are many junctions and curves that cause drivers to move quickly and severely. During those seconds, it is often difficult for the algorithm to detect the face due to its steering angle. However, the algorithm does respond to a certain range of head turns and is able to quantify the angle and time to relate it to distractions, which can be interesting.

Road driving monitoring tests have been carried out to cover a set of situations that the developed prototype can face, such as variability in brightness, use of accessories by the driver, different seat postures, different subjects, etc. Each variant of the drowsiness detection program has been studied to extract its strengths and weaknesses.

**Figure 19:**
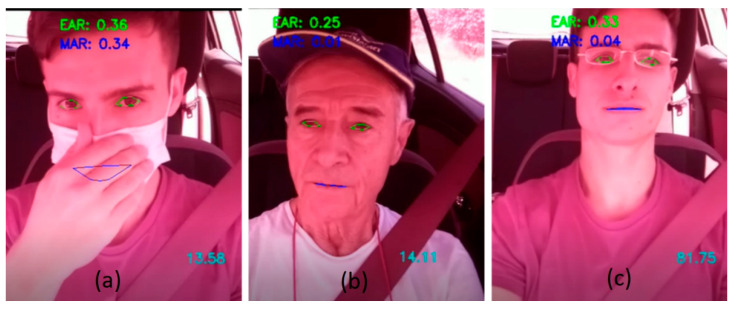
Correct operation with the use of accessories: (**a**) mask, (**b**) cap, (**c**) glasses.

Figure 19a shows a correct facial detection by Haar classifiers, even with partial occlusion of the face due to the use of the mask. It is observed that the coordinates extracted from the eyes are correct. Figure 19b,c show a situation with accessories such as the cap and glasses, and the facial detection is also correct.

Tests in low light conditions at night have been successful due to the infrared LED lamp, which, by eliminating the LDR, allows images of the driver’s face to be captured very effectively throughout the journey.

The variation in facial features between subjects has been considered very relevant in this work, so customised thresholds have been calculated for each of them with the initialisation routine prior to each test. This incorporation has made the algorithm more robust and versatile.

**Figure 20:**
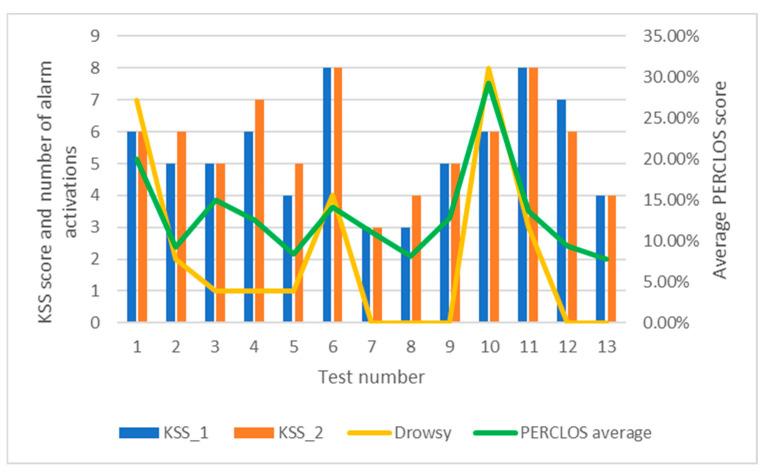
KSS, PERCLOS and drowsiness alarm in the driving tests.

With respect to the Karolinska Sleepiness Scale (KSS), each subject self-assessed a total of two times during each test. The results obtained are shown in Table 3 and Figure 20 below, with the addition of a column for average PERCLOS and another for the activations of the drowsiness alarms.

Figure 20 shows a certain trend between the average PERCLOS scores and the subject’s self-assessments on the KSS scale. However, as this is a subjective scale, it is possible that the subjects’ perceptions do not coincide with the reality of the data. This is the case in test 6, in which the subject was assessed at level 8 but had an average PERCLOS score close to 15%. Despite the relatively low average PERCLOS score, there were four activations of the drowsiness alarm. It may be that the average PERCLOS is low compared to the number of alarm activations, which would mean that there have been periods where there has been enough drowsiness to exceed the 20% PERCLOS threshold and periods where there has been very little drowsiness.

In several of the tests, a proportional correlation between these parameters is followed, as can be seen, for example, in tests 1 and 10. With a larger sample of tests, such a relationship could be determined in order to weigh the relevance of the KSS scale in this algorithm.

## 5. Conclusions

A driving drowsiness detection system designed to be placed in front of the driver, using a Raspberry Pi and a camera, has been built. This system allows the driver to be monitored most of the time, both in light and dark conditions (thanks to an IR lamp), and it is robust against the use of accessories and the driver’s position. It is especially useful for driving on highways or motorways, as drivers become drowsy more easily in these situations, and monitoring is easier because they are looking straight ahead most of the time.

With the tools tested, facial detection through HOG generally provides better results than facial detection through cascades of Haar classifiers. Although face detection is slower, driver monitoring can be performed live, and it is much more robust in light conditions. It is also simpler as it does not require any adjustments to prevent false positives from happening.

The initialisation routine has yielded satisfactory results, with the necessary customisation to correctly assess the indicators in each subject. Table 3 shows the variability in the EAR threshold from 0.17 to 0.28 and in the MAR_threshold from 0.32 to 0.49.

The tests section is the most extensive of this work, and it reflects the results of the algorithm designed for different circumstances. The tests have been carried out on subjects of different genders and ages, ranging from 22 to 58 years old, with different physical characteristics. However, only white Caucasians were included in the sample, which is clearly a bias.

The proposed multi-index methodology shows its value compared to one-dimensional algorithms focusing on index detection, such as EAR. There are different situations in which eye detection becomes more confusing or complicated. A person may close or squint their eyes for a variety of reasons, for example, because they are yawning, want to focus on a distant object, or are in direct sunlight. In the latter case, most people wear sunglasses, whose opacity prevents the eyes from being monitored.

As future work, it is intended to train a specific classifier for the driver’s position that allows detecting the face even if it is not frontally oriented. In order to do this, a set of images of people driving should be used, and in addition to faces appearing from the front, faces turned to both sides should be included. It is not necessary to have them completely rotated (it would be useless because the detection could work, but the extraction of coordinates would not be good). However, having training images with faces slightly rotated would open a range of possibilities for installing the system inside the car. This would allow it to be placed at an angle with respect to the driver, making the detection system more robust.

## Figures and Tables

**Table 1:** Comparison of different DDD systems.

Type	Description	Examples	Advantages	Disadvantages
Subjective	Level of drowsiness is evaluated through a questionnaire	Karolinska scale (KSS), Epworth scale (ESS)	Simple and low-cost	Individual perception bias. Difficult online application.
Driving	Analysis of the patterns of behaviour of the driver	Angle of rotation of steering wheel, frequent lane shift, vehicle speed	Achievable signals in many vehicles	Road conditions, vehicle type
Driver	Analysis of the facial changes of the driver	Opening degree of the eyes and mouth, position and movement of the head	Non-invasive and based on unavoidable drowsiness physical symptoms	Conditions such as lighting or camera position can alter results
Physiological	Muscular, cerebral and cardiovascular type signals	EEG signals, ECG signals, cardiac activity	Drowsiness symptoms not visible but present	Invasive measuring
Mixed	Combination of several measures	Combine several of the other methods at the same time	Higher quality results by not depending on only one measure	More complex to develop

**Table 2:** Thresholds used for the drowsiness detection algorithm.

Area	Threshold	Definition	Value
	Visual indicators		
Eyes	EAR_thresh	EAR limit below which the eyes are considered to be closed	Adaptive depending on the initialisation routine
Mouth	MAR_thresh	MAR limit above which the mouth is considered to be wide open	Adaptive depending on the initialisation routine where it can be used. If not, it shall be 0.35
Look	Gaze_thresh	Gaze limit above which the gaze is considered to be off centre	0.4
Head	Pitch_thresh	Pitch angle limit to consider the head not centred	180
	Yaw_thresh	Yaw angle limit to consider the head not centred	30
	Roll_thresh	Limit of the roll angle to consider the head not centered	20
	Time indicators		
Eyes	EAR_time_thresh	Time limit for eyes closed below EAR_thresh consecutively	3 s
Mouth	MAR_time_thresh	Time limit to have the mouth open above MAR_thresh consecutively	3 s
Look	Gaze_time_thresh	Time limit for unfocused gaze, according to Gaze_thresh, consecutively	4 s
Head	Pose_time_thresh	Time limit for consecutive distracted head poses	6 s
Eyes	PERCLOS_thresh	Percentage limit of time allowed to keep eyes closed below the EAR_thresh threshold for 60 s	0.2 (20%, equivalent to 12 s)

**Table 3:** Compilation of the subjects’ self-assessments in the KSS tests, together with the average PERCLOS obtained and the activations of the drowsiness alarms.

				Detector		Custom thresholds		PERCLOS score	Alarms activated				
		Sleepiness level (KSS)	Face detection	HOG	Haar Cascade	MAR	EAR	PERCLOS average	Yawn	Somnolent	Asleep	Distracted	Looking away
Test	Test 1	6	92.40%	96.13%	3.87%	0.45	0.23	19.94%	0	7	2	10	1
	Test 2	5 and 6	96.36%	96.70%	3.30%	0.32	0.25	9.24%	1	2	2	12	0
	Test 3	5	81.85%	36.37%	63.63%	0.37	0.20	14.98%	3	1	2	6	1
	Test 4	6 and 7	93.75%	98.13%	1.87%	0.49	0.17	12.53%	2	1	2	3	0
	Test 5	4 and 5	98.22%	94.70%	5.30%	0.35	0.28	8.39%	0	1	0	3	1
	Test 6	8	98.20%	95.12%	4.88%	0.35	0.18	14.19%	3	4	2	3	1
	Test 7	3	98.33%	98.70%	1.30%	0.33	0.22	11.10%	0	0	0	3	2
	Test 8	3 and 4	98.43%	92.30%	7.70%	0.30	0.19	8.19%	0	0	0	9	0
	Test 9	5	95.56%	84.35%	15.65%	0.41	0.22	12.76%	3	0	1	4	3
	Test 10	6	95.07%	95.84%	4.16%	0.30	0.20	29.30%	0	8	5	6	0
	Test 11	8	96.14%	99.67%	0.33%	0.31	0.21	13.61%	5	3	3	9	0
	Test 12	7 and 6	96.55%	94.79%	5.21%	0.47	0.17	9.40%	2	0	2	12	0
	Test 13	4	99.56%	98.57%	1.43%	0.31	0.28	7.76%	1	0	0	2	0

## Data Availability

The original contributions presented in the study are included in the article, further inquiries can be directed to the corresponding author.

## Appendix A

The appendix shows the main data obtained in the driving tests.

TEST 1

The participant is a 23-year-old white female of Caucasian ethnicity with an 8-month-old driver’s licence. She drives almost every day and defines herself as a moderately active person. The night before the test, which started around 6 p.m., she had slept for about 5 h. His sleep habits are irregular, sleeping between 6 and 7 h a day. He reports no history of road accidents.

**Table A1:** Compilation table of data from test 1.

Test	Test 1		
Conditions	Level of drowsiness (assessed by the subject with the KSS index)	6 (Some symptoms of drowsiness) on both occasions	
	Brightness	Average: cloudy and raining	
Results	Face detection rate	92.4%	
	Detector	HOG	96.13%
		Cascades of Haar	3.87%
	Detection speed	Maximum	3.77 fps
		Minimum	2.13 fps
		Average	3.49 fps
	Customised thresholds	MAR	0.45
		EAR	0.23
	PERCLOS score	Average	19.94%
		Maximum	33.33%
	Alarms activated	Yawn	0
		Drowsy	7
		Asleep	2
		Distracted	10
		Distracting glance	1
Comments	There have been some sleepy and drowsy alarms that are consistent with the level of drowsiness on the KSS scale.		

In this test, a 92% face detection rate was achieved, of which 96.13% was achieved with the HOG detector, without having to resort to Haar Cascades to find the face in the image. The average frame rate was 3.49 fps. The thresholds customised by the initialisation routine are 0.45 for the MAR and 0.23 for the EAR, scores that are very well adapted to the test person. The maximum PERCLOS score reached in one minute was 33.33%, which triggered the drowsiness alarm. The PERCLOS score exceeded 20% in 7 of the 20 min of the test. The average, however, was 19.94%, which means that in other periods the driver was more attentive. In addition, there were ten distraction alarms, some related to movements to look to the sides of the vehicle, one gaze distraction alarm and no yawning.

There have been some false positives, usually at times when the driver’s head is turned very much to one side, and the detector is not able to recognise the face. However, false positives have been rare, so it is considered to be a fairly successful test.

The driver states that she wears contact lenses and that this causes excessive dryness of the eyes, which increases the amount of blinking and, therefore, the PERCLOS score, which triggers the drowsiness alarm when it exceeds 20%. In addition, despite a cloudy day, at times when the sun was out, the driver’s reflexes were squinting, which may also increase the PERCLOS score without necessarily meaning drowsiness.

TEST 2

The participant is a 58-year-old white woman of Caucasian ethnicity with a 37-year driving licence. The frequency of driving is daily, albeit for short distances and in towns. Frequent physical activity profile. The test started at around 18:30 p.m., having rested for 8 h the night before. Her sleep habits are fairly regular, sleeping around 8 h a day, although with episodes of wakefulness in the early hours of the morning. She reports no history of road accidents.

**Table A2:** Compilation table of test 2 data.

Test	Test 2		
Conditions	Level of drowsiness (assessed by the subject with the KSS index)	5 (neither alert nor drowsy) and 6 (some symptoms of drowsiness)	
	Brightness	Average: cloudy	
Results	Face detection rate	96.36%	
	Detector	HOG	96.70%
		Cascades of Haar	3.30%
	Detection speed	Maximum	3.75 fps
		Minimum	1.88 fps
		Average	3.49 fps
	Customised thresholds	MAR	0.32
		EAR	0.25
	PERCLOS score	Average	9.24%
		Maximum	28.57%
	Alarms activated	Yawn	1
		Drowsy	2
		Asleep	2
		Distracted	12
		Distracting glance	0
Comments	There were some sleepy and drowsy alarms, but mainly distractions. Face detection was accurate, with no false positives.		

In this test, a 96.36% face detection rate was achieved, so the detectors performed quite well. Of these, 96.7% were performed with the HOG detector. There were no false positives, so the results in terms of face detection are optimal. The average frame rate was 3.49 fps, the same as in the first test. The thresholds customised by the initialisation routine are 0.32 for the MAR and 0.25 for the EAR, scores that are also very well adapted to the test person. The average PERCLOS score was 9.24%, and the maximum one-minute PERCLOS score was 28.57%, which triggered the drowsiness alarm. The 20% PERCLOS was exceeded in only 2 of the 20 min of the test. There were also twelve distraction alarms, some of them related to sideways-looking movements, two sleepiness alarms, no gaze distraction and one yawning alarm.

TEST 3

The participant is a 58-year-old white male, Caucasian, with a 39-year driving licence. The frequency of driving is daily, with different types of journeys. He has a medium physical activity profile. The test started at around 19:30 p.m., having rested for 8 h the night before. His sleep habits are fairly regular, sleeping around 8 h a day. He reports no history of road accidents. The driver wears glasses.

**Table A3:** Compilation table of test 3 data.

Test	Test 3		
Conditions	Level of drowsiness (assessed by the subject with the KSS index)	5 (neither alert nor drowsy) on both occasions	
	Brightness	High: sunny	
Results	Face detection rate	81.85%	
	Detector	HOG	36.37%
		Cascades of Haar	63.63%
	Detection speed	Maximum	3.72 fps
		Minimum	1.77 fps
		Average	2.64 fps
	Customised thresholds	MAR	0.37
		EAR	0.2
	PERCLOS score	Average	14.98%
		Maximum	35.24%
	Alarms activated	Yawn	3
		Drowsy	1
		Asleep	2
		Distracted	6
		Distracting glance	1
Comments	There have been few alarms and some of them erroneous due to the high number of false positives.		

A face detection rate of 81.85% was achieved, of which only 36.37% was achieved with the HOG detector. This means that face detection was costly, and Haar Cascades had to be used in most cases, with a high number of false positives. The results are not very satisfactory with this particular subject. It is possible that these results are due to the distance adopted by the driver from the camera, with detection results worsening as the subject gets further away.

The average processing speed was 2.64 fps, which is lower than the previous ones because the difficulty of detecting a face leads to more processing time. The custom thresholds are 0.37 for the MAR and 0.2 for the EAR.

TEST 4

The participant is a 24-year-old white female of Caucasian ethnicity with Nordic features. She has had her driving licence for 1 year. She drives almost every day and defines herself as a sedentary rather than an active person. The night before the test, which started around 8 p.m., she had slept about 5 h and worked 8 h that day. His sleeping habits are regular, sleeping between 7 and 8 h a day. He reports no history of road accidents.

**Table A4:** Compilation table of test 4 data.

Test	Test 4		
Conditions	Level of drowsiness (assessed by the subject with the KSS index)	6 (some symptoms of drowsiness) and 7 (drowsy, no effort to stay awake)	
	Brightness	High	
Results	Face detection rate	93.75%	
	Detector	HOG	98.13%
		Cascades of Haar	1.87%
	Detection speed	Maximum	3.79 fps
		Minimum	2.12 fps
		Average	3.5 fps
	Customised thresholds	MAR	0.49
		EAR	0.17
	PERCLOS score	Average	12.53%
		Maximum	28.10%
	Alarms activated	Yawn	2
		Drowsy	1
		Asleep	2
		Distracted	3
		Distracting glance	0
Comments	Few alarms have been raised despite the driver’s self-assessment of some drowsiness.		

The detection rate is very positive, especially because it is accompanied by face detection with a HOG of almost 100%. In addition, there are no false positives. The thresholds of the EAR and MAR indicators are well-adjusted to the test subject. The PERCLOS is relatively low for what the driver has considered to be her drowsy state. As mentioned above, it is possible that the self-perception is altered and does not truly reflect the subject’s state, so the driver was probably more likely to be in state number 5 on the KSS scale, neither alert nor drowsy.

TEST 5

The participant is a 22-year-old white male of Caucasian ethnicity who has been driving for 4 years. He drives to work and university every day. His lifestyle is active, doing sports 4–5 days a week. The night before the test, he slept for 7 h and worked for 5 h that day. Just before the test, which started at around 5 p.m., he took a nap of just over half an hour. He reports no history of road accidents.

**Table A5:** Compilation table of test 5 data.

Test	Test 5		
Conditions	Level of drowsiness (assessed by the subject with the KSS index)	4 (rather alert) and 5 (neither alert nor drowsy)	
	Brightness	High: sunny	
Results	Face detection rate	98.22%	
	Detector	HOG	94.70%
		Cascades of Haar	5.30%
	Detection speed	Maximum	3.76 fps
		Minimum	2.00 fps
		Average	3.47 fps
	Customised thresholds	MAR	0.35
		EAR	0.28
	PERCLOS score	Average	8.39%
		Maximum	11.43%
	Alarms activated	Yawn	0
		Drowsy	1
		Asleep	0
		Distracted	3
		Distracting glance	1
Comments	There were hardly any alerts because the driver was in an average state of alertness, concentrating on driving.		

The face detection rate is very successful, exceeding 98%, of which about 95% occurred with HOG. Furthermore, no false positives occurred. Regarding the EAR and MAR thresholds, they have been adjusted to the test subject appropriately; they are 0.35 for MAR and 0.28 for EAR. The PERCLOS is in line with the driver’s self-perception during the test, with an average of 11.43% of the time with eyes closed.

In terms of frame rate, it averaged 3.47 fps, similar to the rest, with the exception of Test 3.

TEST 6

The participant is a 23-year-old white male of Caucasian ethnicity. He has had his driving licence for 3.5 years. He drives on a daily basis, much of it in monotonous environments such as the motorway to work, and defines himself as a sedentary rather than an active person. The night before the test, he had slept about 5 h and worked 8 h that day. His sleep habits are regular, sleeping between 5 and 6 h a day, so he suffers from accumulated fatigue. He reports no history of road accidents.

**Table A6:** Compilation table of test data 6.

Test	Test 6		
Conditions	Level of drowsiness (assessed by the subject with the KSS index)	8 (drowsy, some effort to stay awake) on both occasions	
	Brightness	High: sunny	
Results	Face detection rate	98.20%	
	Detector	HOG	95.12%
		Cascades of Haar	4.88%
	Detection speed	Maximum	3.77 fps
		Minimum	2.11 fps
		Average	3.49 fps
	Customised thresholds	MAR	0.35
		EAR	0.18
	PERCLOS score	Average	14.19%
		Maximum	29.05%
	Alarms activated	Yawn	3
		Drowsy	4
		Asleep	2
		Distracted	3
		Distracting glance	1
Comments	Some sleepy and drowsy alarms occurred due to the drowsy state of the driver. There have also been 3 yawns which have been correctly detected. He was not very distracted, but showed signs of being tired.		

The participant in this test assessed himself at a high level of sleepiness, with a score of 8 out of 9, according to the KSS. The PERCLOS is in line with the driver’s self-perception during the test, with an average of 14.19% of the time with eyes closed and a maximum of 29.05% of the time with eyes closed. All alarms were therefore produced, mainly drowsiness, followed by distractions and yawning.

The face detection rate is also very successful, as in previous cases, exceeding 98%. Of these detections, more than 95% are with the HOG detector, with no false positives. Regarding the thresholds for EAR and MAR, they have been adjusted to the test subject appropriately; they are 0.35 for MAR and 0.18 for EAR.

The frame rate has been average, as in most of the other tests, averaging 3.49 fps.

TEST 7

The participant is a 53-year-old white male of Caucasian ethnicity who has 35 years of driving experience. He is a frequent driver and has an active lifestyle. On the day of the test, he worked 7.5 h and slept about 7 h. He has a stable sleep routine of 7 to 8 h of rest. He reports no history of road accidents.

**Table A7:** Compilation table of test data 7.

Test	Test 7		
Conditions	Level of drowsiness (assessed by the subject with the KSS index)	3 (alert) on both occasions	
	Brightness	High: sunny	
Results	Face detection rate	98.33%	
	Detector	HOG	98.70%
		Cascades of Haar	1.30%
	Detection speed	Maximum	3.76 fps
		Minimum	1.99 fps
		Average	3.52 fps
	Customised thresholds	MAR	0.33
		EAR	0.22
	PERCLOS score	Average	11.10%
		Maximum	19.05%
	Alarms activated	Yawn	0
		Drowsy	0
		Asleep	0
		Distracted	3
		Distracting glance	2
Comments	There were hardly any alarms, only distractions when looking sideways for a certain period of time.		

In this test, the driver perceives himself as alert, which is consistent with the alarms produced during driving. Only a few distractions and one gaze distraction occurred. The average PERCLOS is above 11%, which is an average score that matches his condition.

The face detection rate is as in previous cases, exceeding 98%, of which more than 98% are with the HOG detector, with no false positives. The calculated thresholds for EAR and MAR are 0.22 and 0.33, respectively.

The average speed of the test has remained the same as the rest, averaging 3.52 fps.

TEST 8

The participant is a 57-year-old white male of Caucasian ethnicity. He has had his driving licence for more than 40 years. He is a daily driver, and his lifestyle is active, performing physical work frequently. On the day of the test, he worked 5 h and slept about 6 h. He suffers from alternative insomnia. He reports no history of road accidents.

**Table A8:** Compilation table of test data 8.

Test	Test 8		
Conditions	Level of drowsiness (assessed by the subject with the KSS index)	3 (alert) and 4 (rather alert)	
	Brightness	High: sunny	
Results	Face detection rate	98.43%	
	Detector	HOG	92.30%
		Cascades of Haar	7.70%
	Detection speed	Maximum	3.77 fps
		Minimum	2.11 fps
		Average	3.44 fps
	Customised thresholds	MAR	0.30
		EAR	0.19
	PERCLOS score	Average	8.19%
		Maximum	17.62%
	Alarms activated	Yawn	0
		Drowsy	0
		Asleep	0
		Distracted	9
		Distracting glance	0
Comments	The driver has self-assessed as alert and the results are consistent. Only distraction alerts have occurred and some possibly due to observation at junctions.		

Similar to Test 7, the driver perceives himself as alert. PERCLOS is in line with the driver’s self-perception, with an average of 8.19% of the time with eyes closed and a maximum of 17.62% of the time. Only distraction alarms occurred, although there were 7.

The face detection rate is also above 98%, attributing more than 92% to the HOG detector. Regarding the EAR and MAR thresholds, they have been adjusted to the test subject appropriately, being 0.3 for the MAR and 0.19 for the EAR.

The frame rate has been regular, as in most of the other tests, with an average of 3.44 fps.

TEST 9

The participant is a 55-year-old white woman of Caucasian ethnicity. She has held a driving licence for 37 years. She is a regular daily driver and has an active lifestyle. On the day of the test, she did not work and slept about 7 h. She follows a steady sleep routine of 7–8 h of rest per night. She reports no history of road accidents.

**Table A9:** Compilation table of test data 9.

Test	Test 9		
Conditions	Level of drowsiness (assessed by the subject with the KSS index)	5 (neither alert nor drowsy) on both occasions	
	Brightness	High: sunny	
Results	Face detection rate	95.56%	
	Detector	HOG	84.35%
		Cascades of Haar	15.65%
	Detection speed	Maximum	3.79 fps
		Minimum	1.88 fps
		Average	3.33 fps
	Customised thresholds	MAR	0.41
		EAR	0.22
	PERCLOS score	Average	12.76%
		Maximum	19.76%
	Alarms activated	Yawn	3
		Drowsy	0
		Asleep	1
		Distracted	4
		Distracting glance	3
Comments	There have been some alarms, some mistaken, such as a yawn, which has been mistaken for a smile.		

A 95.56% face detection rate was achieved, of which 84.35% was achieved with the HOG detector, less effective than in some of the previous tests. There were also some false positives. The average PERCLOS score was 12.76%, and the maximum score achieved in one minute was 19.76%. There were several alarms, including 4 for distraction and 3 for yawning.

The average frame rate was 3.33 fps, a slight decrease from previous tests, probably due to false positives. The thresholds customised by the initialisation routine are 0.31 for the MAR and 0.22 for the EAR, scores that are also very well adapted to the test person.

TEST 10

The participant is a 57-year-old white male of Caucasian ethnicity. He has had his driving licence for 38 years. He is a daily long-distance driver, driving a minimum of 100 km per day. His lifestyle is active, and on the day of the trial, he worked 10 h and slept about 6 h. He follows an unstable sleep routine, going to bed late and sleeping only a few hours and intermittently. He reports two traffic accidents, although they were caused by no fault of his own and were unrelated to driving fatigue.

**Table A10:** Compilation table of data from test 10.

Test	Test 10		
Conditions	Level of drowsiness (assessed by the subject with the KSS index)	6 (some symptoms of drowsiness) on both occasions	
	Brightness	High: sunny	
Results	Face detection rate	95.07%	
	Detector	HOG	95.84%
		Cascades of Haar	4.16%
	Detection speed	Maximum	3.79 fps
		Minimum	2 fps
		Average	3.46 fps
	Customised thresholds	MAR	0.30
		EAR	0.20
	PERCLOS score	Average	29.30%
		Maximum	50.00%
	Alarms activated	Yawn	0
		Drowsy	8
		Asleep	5
		Distracted	6
		Distracting glance	0
Comments	There have been quite a number of alarms, with drowsy being the most frequent, followed by distracted and asleep.		

In line with previous tests, a high detection rate has been achieved in this one, reaching 95.07% of facial detection, of which 95.84% is with the HOG detector. Regarding the PERCLOS, the maximum score is very high at 50%, and so is the average score at 29.3%, concluding that the subject is in a state of considerable fatigue during this test. However, in the subjective survey, this was only perceived with some symptoms of drowsiness. Reviewing his answers to the personal form, he is a person whose sleeping habits are irregular, which could cause him to be fatigued most of the time.

The average frame rate has been 3.46 fps. The thresholds customised by the initialisation routine are consistent with its physiognomy, being 0.302 for the MAR and 0.204 for the EAR.

TEST 11

The test subject is the same as in test 6. What changed in this trial was that it was conducted at night, at around 23:30 h.

**Table A11:** Compilation table of test data 11.

Test	Test 11		
Conditions	Level of drowsiness (assessed by the subject with the KSS index)	8 (drowsy, some effort to stay awake) on both occasions	
	Brightness	Very low: at night	
Results	Face detection rate	96.14%	
	Detector	HOG	99.67%
		Cascades of Haar	0.33%
	Detection speed	Maximum	3.76 fps
		Minimum	2.28 fps
		Average	3.54 fps
	Customised thresholds	MAR	0.31
		EAR	0.21
	PERCLOS score	Average	13.61%
		Maximum	31.90%
	Alarms activated	Yawn	5
		Drowsy	3
		Asleep	3
		Distracted	9
		Distracting glance	0
Comments	All alarms except the gaze distraction alarm have been triggered. The driver is in a medium state of drowsiness.		

The special feature of this test is that it is the first one carried out in very low light conditions, at night. The infrared LED lamp has been active during the entire journey due to the intentional removal of the LDR.

It was a very successful test, achieving a detection rate of over 96%, of which almost 100% was with the HOG detector. The driver has been correctly monitored, producing different alarms that correspond to drowsiness, although perhaps not as excessive as the driver himself has self-assessed on the KSS scale. He had an average PERCLOS of 13.61% and a maximum of 31.90%.

The average processing speed is 3.54 fps, which means that it has remained the same as in the tests with high lighting conditions.

TEST 12

The driver in this test is the same as in test 4. However, in this case, the test was carried out at night under low-lighting conditions.

**Table A12:** Compilation table of data from test 12.

Test	Test 12		
Conditions	Level of drowsiness (assessed by the subject with the KSS index)	7 (drowsy, no effort to stay awake) and 6 (some symptoms of drowsiness)	
	Brightness	Very low: at night	
Results	Face detection rate	96.55%	
	Detector	HOG	94.79%
		Cascades of Haar	5.21%
	Detection speed	Maximum	3.77 fps
		Minimum	2.13 fps
		Average	3.48 fps
	Customised thresholds	MAR	0.47
		EAR	0.17
	PERCLOS score	Average	9.40%
		Maximum	18.57%
	Alarms activated	Yawn	2
		Drowsy	0
		Asleep	2
		Distracted	12
		Distracting glance	0
Comments	Mostly distracted driving alarms have occurred. The driver is relatively alert.		

This test was carried out at night, with very low lighting conditions. In the compilation table above, it can be seen how the algorithm performed well, achieving a face detection of 96.55%, with 94.79% using the HOG detector.

For the most part, there have been driver distraction alarms. There was also some yawning. The average PERCLOS was 9.4% and the maximum 18.57%, not indicating too much drowsiness with this parameter. However, the driver did self-assess herself as being somewhat drowsy, which suggests that when it is nighttime, there is a tendency to think of oneself as drowsy.

The average processing speed is 3.48 fps.

TEST 13

The driver is a 36-year-old white male of Caucasian ethnicity. His driving licence is 16 years old. He drives weekly. His lifestyle is active, and on the day of the test, he did not work and slept for about 6–7 h. He follows a stable sleep routine of about 6–7 h per night, although he sometimes has sleep interruptions. He reports no traffic accidents.

**Table A13:** Compilation table of data from test 13.

Test	Test 13		
Conditions	Level of drowsiness (assessed by the subject with the KSS index)	4 (rather alert) on both occasions	
	Brightness	High: sunny	
Results	Face detection rate	99.56%	
	Detector	HOG	98.57%
		Cascades of Haar	1.43%
	Detection speed	Maximum	3.77 fps
		Minimum	2.23 fps
		Average	3.53 fps
	Customised thresholds	MAR	0.31
		EAR	0.28
	PERCLOS score	Average	7.76%
		Maximum	18.57%
	Alarms activated	Yawn	1
		Sleepy	0
		Asleep	0
		Distracted	2
		Distracting glance	0
Comments	The driver has remained in an alert state, detecting only an occasional yawn and two distractions.		

In this test, a high detection rate of almost 100% was achieved almost exclusively with the HOG detector. With regard to PERCLOS, the maximum score is 18.57%, and the average score is 7.76%, so the driver is not drowsy and is in an alert state. His self-assessment seems to correspond to the results obtained.

The average frame rate has been 3.53 fps. The thresholds customised by the initialisation routine are consistent with its physiognomy, being 0.310 for the MAR and 0.278 for the EAR.
